# Voluntary risk mitigation behaviour can reduce impact of SARS-CoV-2: a real-time modelling study of the January 2022 Omicron wave in England

**DOI:** 10.1186/s12916-022-02714-5

**Published:** 2023-01-19

**Authors:** Ellen Brooks-Pollock, Kate Northstone, Lorenzo Pellis, Francesca Scarabel, Amy Thomas, Emily Nixon, David A. Matthews, Vicky Bowyer, Maria Paz Garcia, Claire J. Steves, Nicholas J. Timpson, Leon Danon

**Affiliations:** 1 Population Health Sciences, Bristol Medical School, University of Bristol, Bristol, UK; 2 Department of Mathematics, University of Manchester, Manchester, UK; 3 School of Biological Sciences, University of Bristol, Bristol, UK; 4 The Alan Turing Institute, London, UK; 5 School of Cellular and Molecular Medicine, University of Bristol, Bristol, UK; 6 Department of Twin Research & Genetic Epidemiology, King’s College London, London, UK; 7 MRC Integrative Epidemiology Unit at University of Bristol, Bristol, UK; 8 Department of Engineering Mathematics, University of Bristol, Bristol, UK

**Keywords:** SARS-CoV-2, Omicron variant, Behaviour change, Infectious disease modelling, Scenario modelling, policy, Interventions, Longitudinal cohort studies

## Abstract

**Background:**

Predicting the likely size of future SARS-CoV-2 waves is necessary for public health planning. In England, voluntary “plan B” mitigation measures were introduced in December 2021 including increased home working and face coverings in shops but stopped short of restrictions on social contacts. The impact of voluntary risk mitigation behaviours on future SARS-CoV-2 burden is unknown.

**Methods:**

We developed a rapid online survey of risk mitigation behaviours ahead of the winter 2021 festive period and deployed in two longitudinal cohort studies in the UK (Avon Longitudinal Study of Parents and Children (ALSPAC) and TwinsUK/COVID Symptom Study (CSS) Biobank) in December 2021. Using an individual-based, probabilistic model of COVID-19 transmission between social contacts with SARS-CoV-2 Omicron variant parameters and realistic vaccine coverage in England, we predicted the potential impact of the SARS-CoV-2 Omicron wave in England in terms of the effective reproduction number and cumulative infections, hospital admissions and deaths. Using survey results, we estimated in real-time the impact of voluntary risk mitigation behaviours on the Omicron wave in England, if implemented for the entire epidemic wave.

**Results:**

Over 95% of survey respondents (N_ALSPAC_ = 2686 and N_Twins_ = 6155) reported some risk mitigation behaviours, with vaccination and using home testing kits reported most frequently. Less than half of those respondents reported that their behaviour was due to “plan B”. We estimate that without risk mitigation behaviours, the Omicron variant is consistent with an effective reproduction number between 2.5 and 3.5. Due to the reduced vaccine effectiveness against infection with the Omicron variant, our modelled estimates suggest that between 55% and 60% of the English population could be infected during the current wave, translating into between 12,000 and 46,000 cumulative deaths, depending on assumptions about severity and vaccine effectiveness. The actual number of deaths was 15,208 (26 November 2021–1 March 2022). We estimate that voluntary risk reduction measures could reduce the effective reproduction number to between 1.8 and 2.2 and reduce the cumulative number of deaths by up to 24%.

**Conclusions:**

Predicting future infection burden is affected by uncertainty in disease severity and vaccine effectiveness estimates. In addition to biological uncertainty, we show that voluntary measures substantially reduce the projected impact of the SARS-CoV-2 Omicron variant but that voluntary measures alone would be unlikely to completely control transmission.

**Supplementary Information:**

The online version contains supplementary material available at 10.1186/s12916-022-02714-5.

## Background

Throughout the COVID-19 pandemic, human behaviour has modified and controlled transmission. In the initial epidemic growth phase in early 2020, government-defined social distancing, including school closures and stay-at-home orders, was widely used worldwide to prevent the exponential growth in numbers of cases. However, as the pandemic progressed and personal biosecurity measures have become available, the role of individual choice in using face coverings, rapid home tests and vaccination has become increasingly important.

A challenge for predicting future SARS-CoV-2 burden is accurately capturing the population response to measures and advice. The first impositions of stay-at-home orders were widely adhered to [1, 2]. Behaviours changed rapidly upon the imposition of restrictions but were slower to revert to ‘normal’ once restrictions were lifted, with a high proportion of individuals still practicing biosecurity measures such as wearing face coverings and limiting travel [1]. Such behaviours are influenced by multiple factors including trust in the government, individual perception of risk, media reporting and personal interactions, and the importance of different factors varies over time [3].

Behavioural dynamics, which affect both transmission potential and reporting of cases [4], are increasingly included in infectious disease models, although with varying degrees of detail. In particular, the availability of Google Mobility data [5] during the COVID-19 pandemic has been used as a proxy for changing mobility and contact behaviour in transmission models [6–8]; however, assumptions about future behaviour are still required. Surveys of intended behaviours have the potential to aid future predictions but may overestimate realised behaviour [9].

Here, we report on the results of a rapid, prospective survey of intended behaviours over the 2021 winter festive period. The purpose of the study was to estimate the impact of voluntary behaviours on SARS-CoV-2 transmission and numbers of cases and deaths due to the Omicron SARS-CoV-2 variant, first identified in November 2021. In the UK at the time, there were minimal legal restrictions on behaviours, although due to the Omicron variant, the UK government announced a ‘plan B’ on 8 December 2021, in which face masks were compulsory in indoor public spaces and home working was encouraged.

At the time this work was undertaken in December 2021, there was still considerable uncertainty about the transmission potential and severity of the Omicron variant compared to the previously dominant Delta variant. The rapid growth, with cases due to Omicron doubling every 2 to 3 days, suggested a substantial advantage over other variants [10, 11]. This transmission advantage could be due to an increase in transmission potential and/or immune escape mutations which render immunity from vaccination and previous infections less protective against infection than before [12, 13]. Emerging evidence suggests that infection with Omicron is less likely to lead to severe disease and death [14, 15], although with considerable uncertainty.

## Methods

### Rapid survey of social contacts and risk mitigation behaviour during December 2021

We developed an online survey about plans for Christmas 2021 to fill the gap in social contact and behavioural data. The survey covered the festive period from 20 December 2021 to 2 January 2022 and included questions about planned face-to-face interactions, numbers of households meeting indoors, vaccination and risk mitigation behaviours (complete list of questions in Additional file 1). The survey was advertised to the participants of three longitudinal cohorts: the Avon Longitudinal Study of Parents and Children (ALSPAC) [16–18], TwinsUK [19, 20] and the COVID Symptom Survey (CSS) Biobank [21]. TwinsUK and the CSS Biobank were managed by the same team and were treated as one combined cohort for this study.

ALSPAC is an intergenerational prospective birth cohort from the southwest of England. The study recruited 14,541 pregnant women with expected dates of delivery between 1 April 1991 and 31 December 1992 in the former county of Avon and has followed the women, their partners and children since. Full details of the cohort and study design have been described previously [16–18] and are available at www.bristol.ac.uk/alspac. The ALSPAC survey was deployed using Microsoft Forms. The survey was an anonymous, standalone survey and data were not linked to any other data on participants. The survey link went live on 9 December 2021 and was active until 22 December 2021. Participants of ALSPAC were invited to participate via a link in the annual newsletter which went to participants on 15 December 2021 and via social media posts, although anyone with the link could complete the survey.

TwinsUK is a UK registry of volunteer twins in the United Kingdom, with about 14,000 registered twins [19, 20]. The COVID Symptom Study (CSS) Biobank is a longitudinal study run by researchers at King’s College London with approximately 12,000 participants [21]. The TwinsUK/CSS Biobank survey was implemented in REDCap, accessible via an anonymous link advertised in the Christmas newsletter. The survey link was active from 15 to 20 December 2021.

Data from the surveys were analysed in R version 4.01. We calculated descriptive statistics by age, calculating the mean and 95% binomial confidence intervals. We used a logistic regression model to explore associations between risk mitigation behaviours.

### Modelling approach

We used an individual-based disease model based on social contact data, validated against the early growth of SARS-CoV-2 in England in 2020 [8]. The basic premise behind the approach is that we calculate a distribution of individual reproduction numbers for the entire population, based on individuals’ reported social contacts.

Say individual *i* has *k*_*i*_ social contacts on a given day. Each *k*th social contact involves *n*_*k*_ other individuals and it lasts for a time *d*_*k*_, which acts to weight the number of contacts. Their personal individual reproduction number (i.e. the number of secondary cases they generate) is given by

$${R}_i=\tau \sum_{k=1}^{k_i}{\left[ SAR\right]}_k{n}_k{d}_k,$$

where [*SAR*]_*k*_ is the Omicron-specific secondary attack rate (proportion of contacts that result in secondary infection) for the setting of the social contact, either household or non-household and *τ* is a constant calibrated to the reproduction number *R*_0_ = 7 for the Delta variant (i.e. with Delta-specific secondary attack rates) in the absence of vaccination or natural immunity. We used social contact data from the social contact survey (SCS) [22, 23] and secondary attack rates estimated by UKHSA from positive tests in contacts named to NHS Test and Trace [14]. We use [*SAR*]_*k*_ to weight the type of contact (e.g. household contacts are more “intimate” than non-household ones) but assume that, for a given type of contact, all individuals have the same infectivity and susceptibility.

To calculate the population-level reproduction number from the individual reproduction numbers, we assume proportionate mixing between individuals, i.e. that the probability of contacting individual *j* is proportional to their number of contacts over the total number of contacts in the population, *R*_*j*_/∑_*j*_*R*_*j*_. The population-level reproduction number therefore scales with the square of the individual-level reproduction numbers:

$${R}_t\sim \frac{\sum_j{\left({R}_j\right)}^2}{\sum_j{R}_j}.$$

We use the individual reproduction numbers to calculate the cumulative numbers of cases, hospital admissions and deaths. We use the notation *σ*_*j*_ to denote the probability that individual *j* does not get infected during the ensuing epidemic wave. *σ*_*j*_ depends on the susceptibility of individual *j* but also on the infectiousness of all other individuals and the probability that they do/do not get infected; therefore, there is no closed form solution for calculating *σ*_*j*_. Following [24], *σ*_*j*_ can be shown to be

$$\log {\sigma}_j=-{R}_j\frac{\sum_k{R}_k\left(1-{\sigma}_k\right)}{\sum_k{R}_k}.$$

As there is no closed form solution for calculating *σ*_*j*_, it is calculated by iteration, starting with *σ*_*j*_ = 0.5 for all *j*s and recalculating all final sizes, repeating until the estimates converge.

The cumulative number of cases is calculated from the individual *σ*_*j*_s by multiplying by an individual-specific weight *w*_*j*_ based on the representativeness of the social contact survey that is used for the model. The total number of cases is ∑_*j*_*w*_*j*_(1 − *σ*_*j*_). The number of deaths is calculated from the number of cases, multiplied by the age-specific infection fatality rate, ∑_*j*_*w*_*j*_(1 − *σ*_*j*_)*μ*_*j*_.

### Modelling vaccination and natural immunity

We capture vaccination using the vaccination line list data provided by UKHSA on 26 November 2021. We aggregated the data to calculate the proportion by age that had received a single or double dose of each of the main available vaccines in England (AstraZeneca or Pfizer/Moderna), and a booster dose, leading to five categories. We estimated the proportion of individuals by age with immunity from a natural infection using the Pillar 2 data of test positive cases, assuming that 50% of infections were identified as cases. Therefore, we model the population using eight categories of vaccine/immune status: five vaccination states, immunity from vaccination and a natural infection, immunity from natural infection only and no immunity/unprotected. We made the simplifying assumption for the initial conditions that vaccine and infection status were independent—the proportions of each age group falling within the eight categories were calculated using data from 26 November 2021 (see Additional file 1, Table S1). We assumed immunity from vaccination and natural infection to provide superior protection against severe disease than vaccination alone.

The effect of vaccination is incorporated into the model via three mechanisms: by reducing the probability that an individual is infected (reduced susceptibility), reducing the probability that the individual will transmit to others (reduced transmissibility) and reducing the risk of severe disease and death. We use UKHSA estimates of vaccine effectiveness for the Delta variant [15] and use the approach in [25] to scale the effectiveness against infection and transmission down by taking the *v*^*th*^ power of the effectiveness expressed as a proportion—therefore, an effectiveness of 80% against Delta becomes 41% against Omicron [25]. We use a different exponent for the reduction in effectiveness against infection and against death.

The individual reproduction number is modified by vaccination by reducing the probability of transmission

$${R}_i^{(v)}=\left(1-{\varepsilon}_t^{(v)}\right)\tau \sum_{k=1}^{k_i}{\left[ SAR\right]}_k{n}_k{d}_k,$$

where ${\varepsilon}_t^{(v)}$ is the vaccine effectiveness against transmission for vaccine/immunity state *v*.

The population-level reproduction number is formed of all vaccine states

$${R}_t^{vac}\sim \frac{\sum_i\left(\sum_v{\alpha}_i^{(v)}{R}_i^{(v)}{r}_i^{(v)}\right)}{\sum_i{R}_i},$$

where ${\alpha}_i^{(v)}$ is the probability that individual *i* is in vaccination state *v* , such that ${\sum}_v{\alpha}_i^{(v)}=1\kern0.5em$and ${r}_i^{(v)}$ is the ‘receiving’ risk of infection,

$${r}_i^{(v)}=\tau \left(1-{\varepsilon}_s^{(v)}\right)\ \sum_{k=1}^{k_i}{\left[ SAR\right]}_k{n}_k{d}_k,$$

with ${\varepsilon}_s^{(v)}$ being the vaccine effectiveness against infection for vaccine state *v* and *k* a constant calibrated to the initial reproduction number without vaccination.

The final size calculations are also modified by the action of the vaccine,

$${\sigma}_i^{(v)}=\exp \left(-{r}_i^{(v)}{\uptheta}_i\right),$$

where

$${\uptheta}_i=\frac{1}{\sum_j{R}_j}\sum_j\sum_v{\alpha}_j^{(v)}{R}_j^{(v)}\left(1-{\sigma}_j^{(v)}\right).$$

The cumulative number of cases is calculated as

$$cases=\sum_j{w}_j\sum_v{\alpha}_j^{(v)}\left(1-{\sigma}_j^{(v)}\right).$$

The cumulative number of hospital admissions is calculated with the individual-specific hospital admission rate *h*_*j*_ as:

$$hospital\ admissions=\sum_j{h}_j{w}_j\sum_v{\alpha}_j^{(v)}\left(1-{\sigma}_j^{(v)}\right).$$

Finally, the cumulative number of deaths is calculated using the individual-specific mortality admission rate *μ*_*j*_ as:

$$deaths=\sum_j{\mu}_j{w}_j\sum_v{\alpha}_j^{(v)}\left(1-{\sigma}_j^{(v)}\right).$$

### Risk mitigation measures

This framework allows us to model risk mitigation measures at an individual level. In the model, an individual is associated with a probability of exhibiting risk mitigation behaviours, according to age, and determined by survey responses. For each model iteration, an individual is determined to practice that risk mitigation measure or not, based on a random number draw. For example, in persons aged 30–39, 67% report limiting in-person visits to shops, 59% report using a face mask, 51% report avoiding public transport, 47% report working from home and 81% report using home testing kits. So, for a single model run for an individual aged 35, we draw a random number, say *rand*_1_ = 0.69, in which case this individual would not limit visits to shops or public transport use, or use a face mask, or work from home, but would use home testing kits. We assume a single number determines all risk mitigation measures for an individual, rather than choosing each of them independently, reflecting the evidence that some individuals tend to be more cautious than others in all their activities.

Contact tracing is applied to symptomatic cases only (where the probability of symptoms is determined by the age of the individual), implemented by reducing the number of secondary cases by a proportion *CTF*, determined from the NHS Test and Trace statistics as approximately (proportion of cases reached and asked to provide details of recent close contacts) × (proportion who provided details for one or more close contact) × (proportion of contacts reached within 24 h) × (proportion of close contacts reached and asked to self-isolate) [26].

Lateral flow testing of asymptomatic cases is implemented in a similar way to contact tracing but originating from asymptomatic cases. Individuals were given an age-specific probability of using home testing kits based on the results of the ALSPAC survey. If a home testing kit was used and infection was identified, the number of secondary cases is reduced by the same proportion as for contact tracing, *CTF*. The sensitivity of lateral flow testing was taken as *s*_*L*_ = 50% [27].

Mask wearing was implemented by reducing the probability of transmission by a proportion *CS*. Our estimate for the current impact of mask-wearing on transmission is less than 25%. In a 2020 systematic review, Chu et al. reported a smaller risk reduction for face mask use in non-healthcare settings compared to for healthcare settings and a smaller reduction for single layer face masks as opposed to respirators and surgical masks [28, 29].

Working from home, limiting in-person shopping and avoiding public transport were implemented by eliminating contacts reported as occurring at work/in shops/on public transport. If a contact did not take place, we set *n*_*k*_ = 0 for that interaction. In addition, we simulated school holidays over Christmas by removing all contacts for children under 18 years old with “school” listed as the context, as these were assumed not to take place during the winter holidays.

### Changes to disease severity

We investigate four severity scenarios to illustrate the potential impact of the Omicron variant: (A) moderate severity and reduced vaccine effectiveness: a 20% reduction in mortality rates associated with Omicron infection and a reduction in vaccine effectiveness against severe disease compared to the Delta variant (*μ*_Ο_ = 0.8, *v*_*I*_ = 4, *v*_*D*_ = 4); (B) low severity and reduced vaccine effectiveness: a 50% reduction in mortality rates associated with Omicron infection and a reduction in vaccine effectiveness against severe disease compared to the Delta variant (*μ*_Ο_ = 0.5, *v*_*I*_ = 4, *v*_*D*_ = 4); (c) moderate severity and no reduction in vaccine effectiveness: a 20% reduction in mortality rates associated with Omicron infection with no reduction in vaccine effectiveness against severe disease (*μ*_Ο_ = 0.8, *v*_*I*_ = 4, *v*_*D*_ = 1); (d) low severity and no reduction in vaccine effectiveness: a 40% reduction in mortality rates associated with Omicron infection with a small reduction in vaccine effectiveness against severe disease (*μ*_Ο_ = 0.5, *v*_*I*_ = 4, *v*_*D*_ = 2).

### Model implementation

The model was written in R version 4.01. The population of individuals was simulated 100 times and results aggregated. A summary of parameter values and interpretations is given in Table 1. The model code is available at https://github.com/ellen-is/Reckoners-Xmas21 [32].

**Table 1:** Parameter values used in the model

Parameter name/interpretation/symbol		Parameter estimates/ranges and sources
Secondary attack rate [*SAR*]	Delta, household	10.3% (10.1–10.5%) [14]
	Delta, non-household	3.0% (2.8–3.2%) [14]
	Omicron, household	15.8% (14.3–17.5%) [14]
	Omicron, non-household	8.7% (7.5–10.0%) [14]
Vaccine effectiveness against infection with the Delta SARS-Cov-2 variant	AZ1 (received 1 dose of the Oxford AstraZeneca vaccine)	30%
	AZ2 (received 2 doses of the Oxford AstraZeneca vaccine)	60% [15]
	PF1 (received 1 dose of the Pfizer vaccine)	30%
	PF2 (received 2 doses of the Pfizer vaccine)	80% [15]
	Booster (received any combination of three vaccine doses)	60% [15]
	Natural immunity (with SARS-CoV-2, no vaccination)	50%
	Vaccinated and natural immunity (any combination of vaccinations and infection with SARS-CoV-2)	50%
	Unprotected (unvaccinated and no prior SARS-CoV-2 infection)	0%
Vaccine-associated reduction in transmission of the Delta SARS-Cov-2 variant	AZ1	45% [30]
	AZ2	70% [15]
	PF1	45% [30]
	PF2	84% [15]
	Booster	90% [15]
	Natural immunity	65%
	Vaccinated and natural immunity	65%
	Unprotected	0%
Vaccine-associated reduction in severe disease (hospital admission and death) due to the Delta SARS-Cov-2 variant	AZ1	80%
	AZ2	94% [15]
	PF1	85%
	PF2	97% [15]
	Booster	96% [15]
	Natural immunity	94%
	Vaccinated and natural immunity	99.5%
	Unprotected	0%
Baseline mortality rate (per 1000 SARS-CoV-2 infections in the absence of vaccination)	5-year age groups {0–4, 5–9, 10–14, 15–17, 18–19, 20–24, 25–29, 30–34, 35–39, 40–44, 45–49, 50–54, 55–59, 60–64, 65–69, 70–74, 75–79, 80+}	{0.0, 0.0, 0.0, 0.2, 0.3, 0.4, 0.8, 1.2, 2, 2, 4, 10, 16, 34, 54, 86, 124, 192, 192, 192} [31]
Hospital admission rate (per 1000 SARS-CoV-2 infections in the absence of vaccination)	5-year age groups {0–4, 5–9, 10–14, 15–17, 18–19, 20–24, 25–29, 30–34, 35–39, 40–44, 45–49, 50–54, 55–59, 60–64, 65–69, 70–74, 75–79, 80+}	{1 1 1 1 2 5 10 16 23 29 39 58 72 102 117 146 177 180 200 200} [31]
Exponent for reduction in vaccine effectiveness against infection, *v*_*I*_		Scenario A/B/C/D: 4/4/4/4
Exponent for reduction in vaccine effectiveness against death, *v*_*D*_		Scenario A/B/C/D: 4/4/1/2
Relative reduction in mortality rate for Omicron versus Delta variants, *μ*_Ο_		Scenario A/B/C/D: 0.2/0.5/0.2/0.5
Lateral flow sensitivity		50% [27]
Contact tracing effectiveness, *CTF*		25% [26]
Face mask effectiveness		25% [29]

## Results

### Risk mitigation behaviour

The ALSPAC and TwinsUK/CSS Biobank surveys received 2686 and 6155 responses respectively. Most respondents (78% and 88% respectively) were aged between 30 and 69 years of age (Table 2). The vast majority of respondents to the ALSPAC survey (96%) were ALSPAC participants.

**Table 2:** Number of responses to the 2021 Christmas survey run by ALSPAC and TwinsUK/CSS Biobank

Age group	ALSPAC	Twins UK/CSS Biobank
Under 30	406	221
30–39	231	1348
40–49	22	1338
50–59	592	1348
60–69	821	1368
70–79	56	476
80 +	0	52
Prefer not to say	0	4

**Fig. 1:**
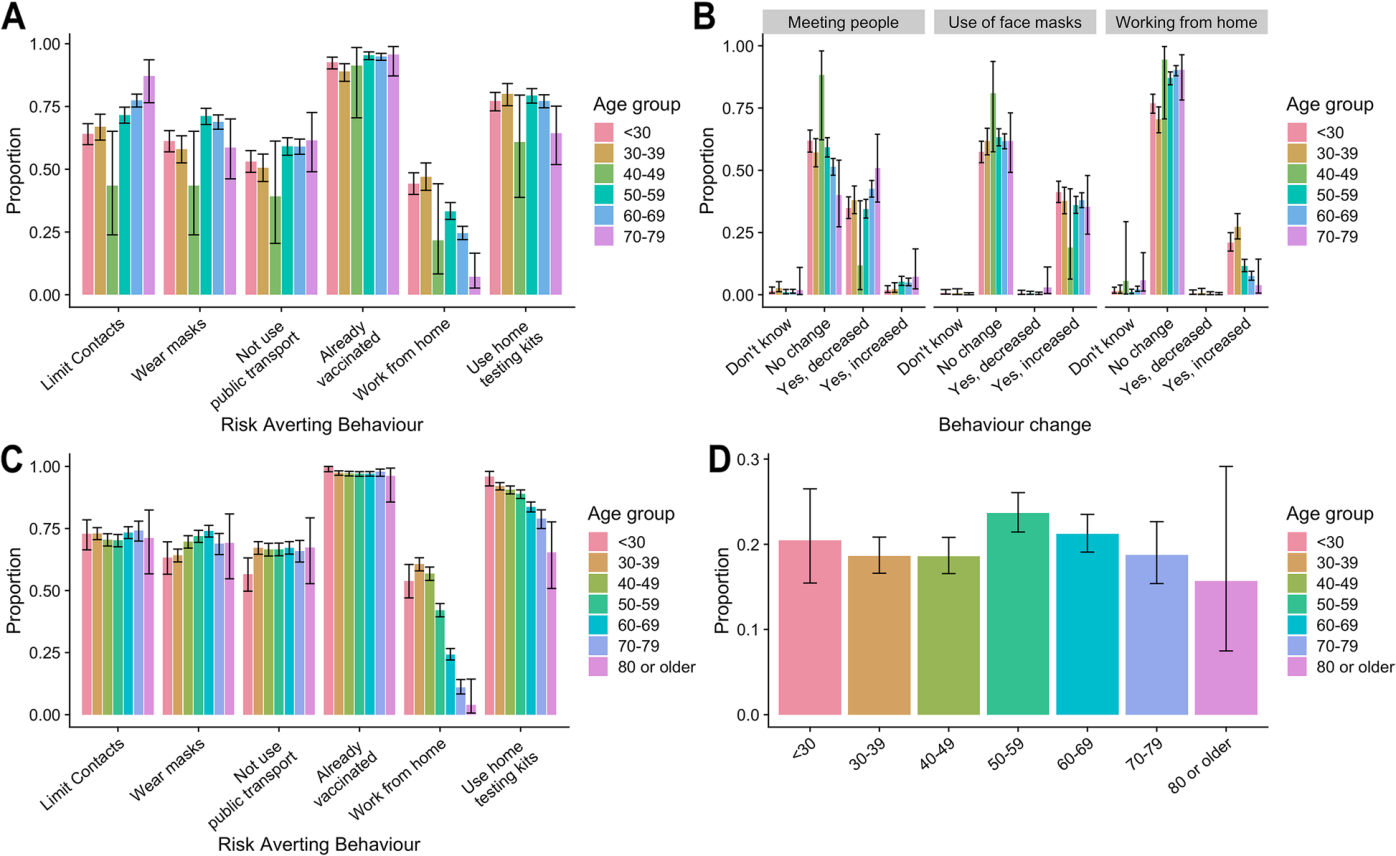
Survey responses from ALSPAC (Avon Longitudinal Survey of Parents and Children) and TwinsUK/CSS Biobank. **A** The proportion of ALSPAC respondents (*N* = 2686) by age group reporting risk mitigation measures during the period 20 December 2021 to 2 January 2022 inclusive. **B** The proportion of ALSPAC respondents who changed their behaviour (meeting people, use of face masks, working from home) due to the announcement of “plan B”. **C** The proportion of TwinsUK/CSS Biobank respondents (*N* = 6155) by age group reporting risk mitigation measures during the period 20 December 2021 to 2 January 2022 inclusive. **D** The proportion of TwinsUK/CSS Biobank respondents who changed their behaviour due to the announcement of “plan B”. The confidence intervals are 95% binomial confidence intervals

The general patterns of risk mitigation measures were similar in both surveys, although TwinsUK/CSS Biobank participants reported slightly higher levels of risk mitigations than ALSPAC participants (Fig. 1A, C and figure S1 in Additional file 1). In both surveys, a high proportion of respondents (over 95%) reported some risk mitigation behaviours. The most frequently reported was getting vaccinated or boosted. Overall, 94% (93%, 95%) of ALSPAC and 97% of TwinsUK/CSS Biobank participants reported that they had or planned to get fully vaccinated, with minimal differences between age groups. Using home testing kits was second to vaccination: 78% (76%, 79%) of ALSPAC participants and 88% of TwinsUK/CSS Biobank participants planned to use lateral flow testing kits before meeting friends and family. There was a decline in the use of home testing kits with age in the TwinsUK/CSS Biobank data.

Face mask use was reported by 66% (65%, 68%) of ALSPAC and 70% of TwinsUK/CSS Biobank participants (Fig. 1A, C). Of the respondents that reported planning to use a face mask, 64% said that “plan B” had no impact on face mask use, and 36% said that use would increase due to “plan B” (Fig. 1B). In both surveys, 72% (70%, 74%) of respondents reported planning to limit contacts; nearly half of ALSPAC respondents who reported this said it had been affected by the announcement of “plan B”. Up to 25% of respondents in the TwinsUK/CSS Biobank survey reported altering their behaviour due to “plan B”, with the highest levels of change reported in 50–59 year olds (Fig. 1D). There were differences in reported use with age and gender; see Additional file 1.

### Omicron SARS-CoV-2 variant in England

**Fig. 2:**
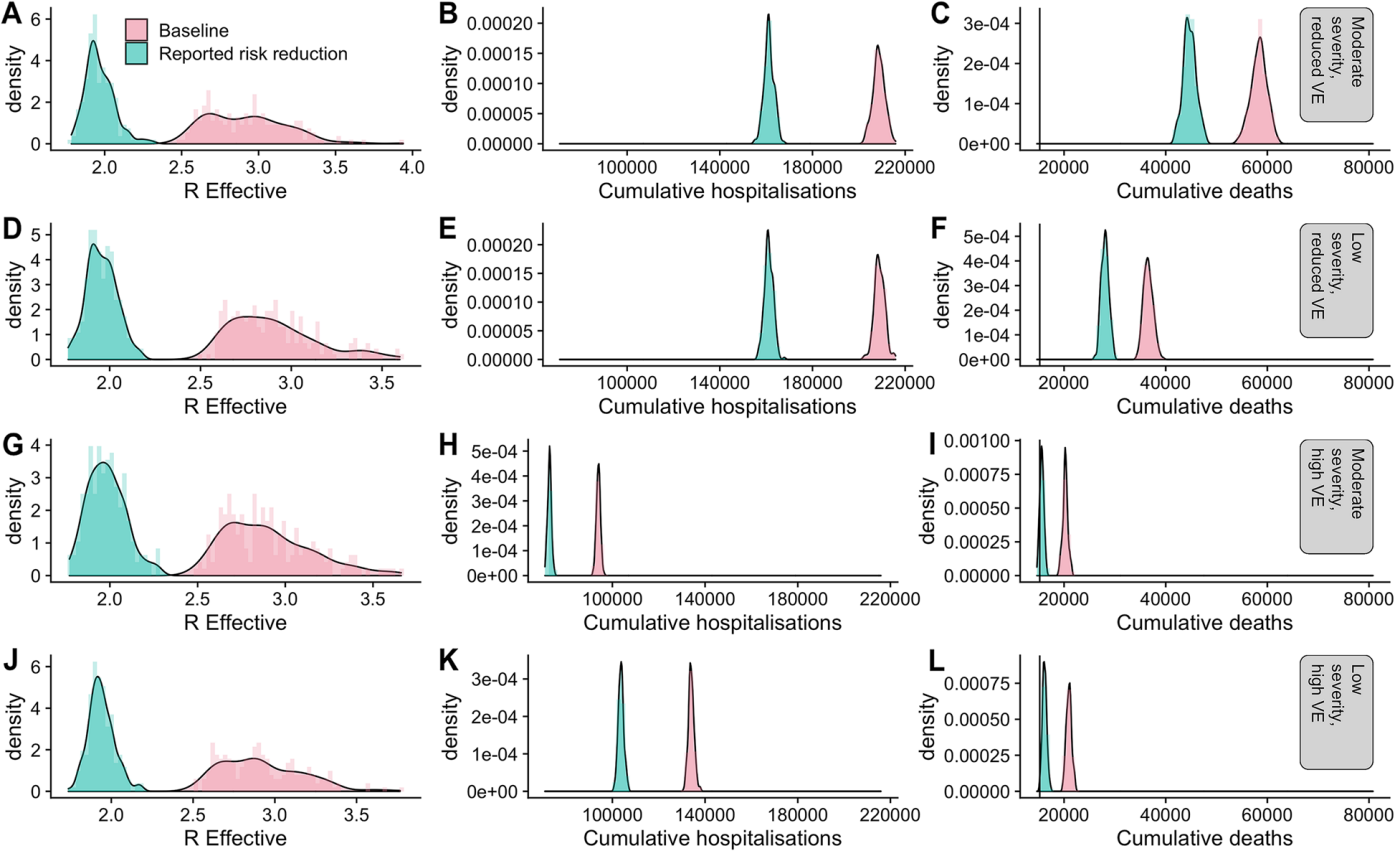
The estimated size of the effective reproduction number (**A**, **D**, **G**, **L**), cumulative hospital admissions (**B**, **E**, **H**, **K**) and cumulative deaths (**C**, **F**, **I**, **L**) with and without reported risk mitigation measures. **A**–**C** With a 20% reduction in severity associated with Omicron relative to Delta. **D**–**F** With a 50% reduction in severity associated with Omicron relative to Delta. **G**–**I** With a 20% reduction in severity associated with Omicron relative to Delta and assuming that vaccine effectiveness against severe disease is not reduced with Omicron infection. **J**–**L** With a 50% reduction in severity associated with Omicron relative to Delta and assuming that vaccine effectiveness against severe disease is marginally reduced with Omicron infection. Vaccine distribution as of 26 November 2021

Using the individual-based model, we estimate that the effective reproduction number for the Omicron variant is 2.9 (95% CI 2.5, 3.5) (Fig. 2A), compared to unity for the Delta variant. The increase in the reproduction number is due to the increase in transmission potential and decrease in vaccine effectiveness and is unaffected by assumptions about disease severity or vaccine effectiveness against severe disease (Fig. 2D, G).

In an unconstrained epidemic, this reproduction number translates to approximately 35 (95% CI 34, 36) million infections. The impact on hospital admissions and deaths is dependent on the relative severity of the Omicron variants compared to the Delta variant. With a 20% reduction in severity relative to the Delta variant, these numbers would be higher: 59,000 (95% CI 56,000–62,000) deaths (Fig. 2A–C). With 50% reduction in severity relative to the Delta variant leads to an estimated 225,000 (95% CI 216,000–236,000) hospital admissions and 44,000 (95% CI 42,000–46,000) deaths (Fig. 2E, F). With a 20% reduction in severity relative to the Delta variant, and no reduction in vaccine effectiveness against severe disease, the estimated cumulative hospital admissions is 110,000 (95% CI 102,000–120,000) and the estimated cumulative number of deaths 20,000 (95% CI 19,000–21,000) (Fig. 2G–I). Finally, with a 50% reduction in severity relative to the Delta variant, and a smaller relative reduction in vaccine effectiveness against severe disease, the estimated cumulative hospital admissions is 94,000 (95% CI 92,000–96,000) and the estimated cumulative number of deaths 13,000 (95% CI 12,000–13,000).

### Impact of voluntary risk mitigation behaviours on the Omicron SARS-CoV-2 variant epidemic in England

With the high reported risk mitigation measures, seen across two cohort studies, we estimate that the Omicron effective reproduction number in England could be reduced to 1.9 (95% CI 1.8–2.2). This reduced reproduction number equates to a 25% reduction in the number of infections to approximately 26 (95% CI 25–27) million cumulative infections, and a similar reduction in cumulative deaths to 34,000 (95% CI 32,000–35,000) (Fig. 2C), under the assumption of 50% reduction in both severity of Omicron relative to Delta and 50% reduction in vaccine effectiveness against severe disease relative to Delta. Since conducting this analysis, the actual number of deaths that occurred between 26 November 2021 and 1 March 2022 was 15,208.

Although relative severity is central to the absolute numbers of hospital admissions and deaths, the relative reduction due to risk mitigation behaviours is consistently between 20 and 25%.

**Fig. 3:**
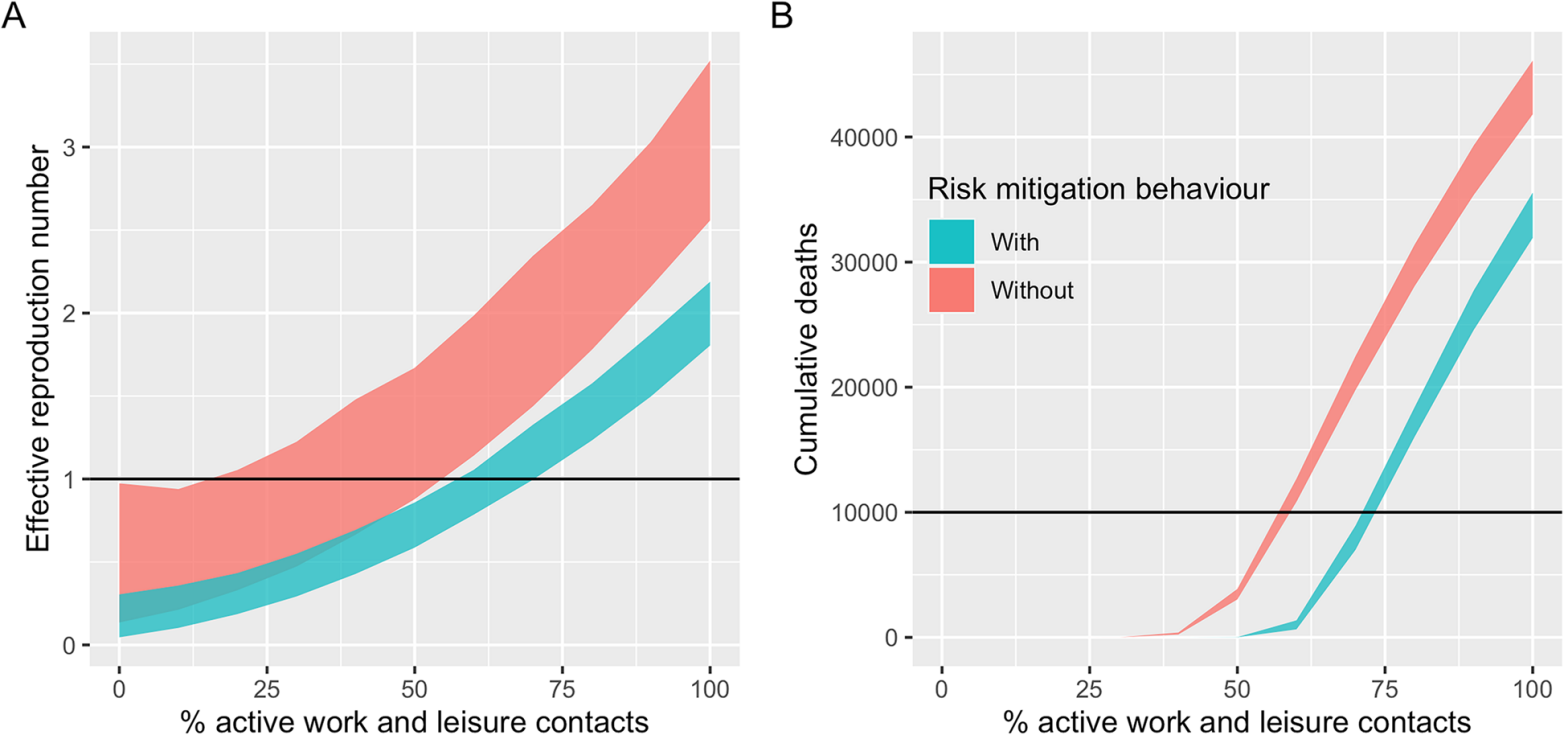
**A** The effective reproduction number with and without risk mitigation behaviour. **B** The cumulative number of deaths with and without risk mitigation behaviour

Figure 3 illustrates how the effective reproduction number and cumulative number of deaths depend on the percentage of normal work and leisure contacts that take place. Without additional risk mitigation behaviours, over half of work and leisure contacts would need to be prevented to achieve a reproduction number of less than 1.

## Discussion

Data here suggest that a high proportion of people intended to practice risk mitigation behaviours to prevent the spread of COVID-19 during the Christmas 2021 period (from 20 December 2021 to 2 January 2022), with approximately 80% reporting using home testing kits. We estimate that these realistic risk-mitigating behaviours, including mask-wearing and regular home testing, could lead to a 40% reduction in the Omicron effective reproduction number and a 28% reduction in cumulative numbers of deaths. Nonetheless, these intended risk mitigation behaviours are not sufficient to reduce the effective reproduction number below one, suggesting that epidemic control of Omicron cannot rely solely on self-imposed restrictions, and a further reduction in social contacts of 25–50% is required for control. Survey results also suggest that announcements of ‘Plan B’ restrictions had limited behavioural impact in our sample and would therefore have limited epidemiological impact. Under our baseline scenario, we estimated approximately 50,000 further deaths due to Omicron alone, which is a 30% increase in the total number of deaths in England between March 2020 and December 2021.

This study was conducted in late 2021, one of a number of models presented at SPI-M-O that explored scenarios for the January 2022 SARS-CoV-2 ‘Omicron’ wave [33, 34]. Scenario modelling, as opposed to prediction or forecasting, is used to understand epidemic drivers such as vaccine coverage, the impact of variants or population behaviour change [35]. With our individual-based approach, we were able to quantify the impact of ‘plan B’ and voluntary behaviour change. Our approach is less computationally intensive than dynamic models, and by using theoretical results, we provide a fast, transparent and intuitive understanding of how behaviour translates to hospital admissions and deaths. A drawback of our approach is that it is not dynamic, so we cannot capture the ongoing effects of vaccine waning, vaccine rollout or changing behaviours. We also cannot estimate trajectories of cases and deaths. The main source of uncertainty in our modelled estimates is uncertainty in the severity of disease, both intrinsic severity (the probability of severe disease in a naïve, unvaccinated individual) and the probability of severe disease in individuals with prior immunity that suffer breakthrough infections.

Our results use surveys of intended behaviours to modulate the transmission potential of SARS-CoV-2. Other approaches for incorporating behaviour into models include using mobility data, such as Google Mobility [5], social network and mobile phone data [36, 37], which have been used during the COVID-19 pandemic. Such routinely collected, geolocated data can fill data gaps from under or absent sampling, for example before other systems are established. Each source of behavioural data offers a different perspective, with varying biases and representativeness [38]. A comparative study showed that the COVID-19 reproduction number was overestimated by mobility data but underestimated by survey data; in general, survey data provided more reliable estimates of transmission changes than mobility data [39].

Furthermore, self-reported future intentions may not reflect actual behaviour. A survey conducted during the pandemic found that while 70% of individuals intended to fully self-isolate with COVID-19 symptoms, only 50% actually did [9]. Underreporting of other risk mitigation measures is less clear: 70% of our cohorts intended to use face coverings, which is less than measured use [40]. Eighty percent of our cohorts intended to use home testing kits before seeing family and friends, which is greater than the 40% [41] and 50% [42] of individuals who reported using home testing kits between April 2021 and June 2021. However, home testing kit use increased by 63% in December 2021, compared to the rest of the year [43], suggesting that intended use was broadly reflective of actual use. Risk mitigation behaviour also varies with socio-demographic factors such as gender and age [41]. While established longitudinal cohorts are designed to be broadly representative, they nevertheless suffer from respondent bias with over-representation of females and higher socioeconomic status [18]. Our surveys did not include many children or young adults, and ALSPAC participants are mostly resident around Bristol. There are likely to be additional unmeasured biases in the surveys and understanding how risk mitigation behaviour varies by age, ethnicity and socioeconomic status would be valuable for improved characterisation of the epidemiology in different communities.

## Conclusions

This study was used in real-time to quantify the necessity of additional restrictions over-and-above voluntary measures. This work demonstrates the power of utilising existing longitudinal cohorts for rapid elicitation of behavioural information in combination with population-levels transmission models. Fostering such links improves the accuracy and utility of disease models used for public health action and decision making especially for rapidly spreading infections.

## Supplementary Information

**Additional file 1: Supplementary Table S1.** Model input values. Percentage of individuals by age group by vaccine and immune status, assuming 50% case ascertainment as of 26 November 2021. Section 2: Statistical analysis of survey results. Section 3: Sensitivity analysis of model output to key parameters. Section 4: Survey questions from the ALSPAC survey. **Figure S1.** Results from the logistic regression models from the two prospective surveys. **Figure S2.** Sensitivity of the model output to four model parameters. **Figure S3.** Exploration of the sensitivity of model output to four parameters.

## Data Availability

The social contact survey data are available at [23]. The informed consent obtained from ALSPAC participants does not allow the survey data to be made freely available through any third party maintained public repository. However, data used for this submission can be made available on request to the ALSPAC Executive. The ALSPAC data management plan describes in detail the policy regarding data sharing, which is through a system of managed open access. Full instructions for applying for data access can be found here: http://www.bristol.ac.uk/alspac/researchers/access/. The ALSPAC study website contains details of all the data that are available (http://www.bristol.ac.uk/alspac/researchers/our-data/). Similarly, TwinsUK/CSS Biobank data access policy is available here: https://twinsuk.ac.uk/resources-for-researchers/access-our-data/. The model code and minimal aggregate data for running the model are available at https://github.com/ellen-is/Reckoners-Xmas21 [32].

## Abbreviations

ALSPAC: Avon Longitudinal Survey of Parents and Children
CSS: COVID Symptom Survey
NHS: National Health Service
SARS-CoV-2: Severe acute respiratory syndrome coronavirus 2
SAR: Secondary attack rate
SCS: Social contact survey
SPI-M-O: Scientific Pandemic Influenza Modelling - Operational
UKHSA: United Kingdom Health Security Agency
