# Supplementary material for: Voluntary risk mitigation behaviour can reduce impact of SARS-CoV-2: a real-time modelling study of the January 2022 Omicron wave in England
Source: BMC Med. 2023 Jan 19;21:25. doi: 10.1186/s12916-022-02714-5 (PMC9851586; doi:10.1186/s12916-022-02714-5)
Supplement: Supplementary file 1 — Additional file 1: Supplementary Table S1. Model input values. Percentage of individuals by age group by vaccine and immune status, assuming 50% case ascertainment as of 26 November 2021. Section 2: Statistical analysis of survey results. Section 3: Sensitivity analysis of model output to key parameters. Section 4: Survey questions from the ALSPAC survey. Figure S1. Results from the logistic regression models from the two prospective surveys. Figure S2. Sensitivity of the model output to four model parameters. Figure S3. Exploration of the sensitivity of model output to four parameters. [file 12916_2022_2714_MOESM1_ESM.docx]

Supplementary information for

Voluntary risk-mitigation behaviour can reduce impact of SARS-CoV-2: a real-time modelling study of the January 2022 Omicron wave in England

Ellen Brooks-Pollock^1*^, Kate Northstone^1^, Lorenzo Pellis^2,3^, Francesca Scarabel^2^, Amy Thomas^1^, Emily Nixon^1,4^, David A. Matthews^5^, Vicky Bowyer^6^, Maria Paz Garcia^6^, Claire J. Steves^6^, Nicholas J. Timpson^1,7^, Leon Danon^8^

^1^Population Health Sciences, Bristol Medical School, University of Bristol, BS8 2BN.

^2^Department of Mathematics, University of Manchester

^3^School of Biological Sciences, University of Bristol, BS8 2BN.

^4^The Alan Turing Institute, London, UK

^5^School of Cellular and Molecular Medicine, University of Bristol, BS8 2BN.

^6^Department of Twin Research & Genetic Epidemiology, King’s College London

^7^MRC Integrative Epidemiology Unit at University of Bristol, Bristol, BS8 2BN, UK.

^8^Department of Engineering Mathematics, University of Bristol.

*Ellen.Brooks-Pollock@bristol.ac.uk

Table of Contents

[Model input: table of population immune status 1](#_Toc118730242)

[Statistical analysis of survey results 2](#_Toc118730243)

[Sensitivity analysis 3](#_Toc118730244)

[Survey questions 4](#_Toc118730245)

# Model input: table of population immune status

Supplementary Table S1: Model input values. Percentage of individuals by age group by vaccine and immune status, assuming 50% case ascertainment as of 26 November 2021. Vaccine status was calculated from vaccine line lists from UKHSA, percentage test positive from UKHSA case data and population denominators from ONS.

| Age group | N | AZ1 | AZ2 | PF1 | PF2 | MOD1 | MOD2 | Booster | Any vaccination | Unvaccinated | Test positive | Natural Immunity only (no vaccination) | Unprotected |
| --- | --- | --- | --- | --- | --- | --- | --- | --- | --- | --- | --- | --- | --- |
| 0-4 | 3,299,637 | 0.0 | 0.0 | 0.0 | 0.0 | 0.0 | 0.0 | 0.0 | 0.0 | 100.0 | 5.9 | 11.7 | 88.3 |
| 5-9 | 3,538,206 | 0.0 | 0.0 | 0.0 | 0.0 | 0.0 | 0.0 | 0.0 | 0.0 | 100.0 | 13.2 | 26.5 | 73.5 |
| 10-14 | 3,354,246 | 0.0 | 0.0 | 25.0 | 0.3 | 0.0 | 0.0 | 0.0 | 25.3 | 74.7 | 25.3 | 50.4 | 49.2 |
| 15-17 | 1,831,479 | 0.1 | 0.4 | 54.2 | 9.8 | 0.0 | 0.0 | 0.4 | 64.6 | 35.4 | 25.5 | 45.6 | 43.9 |
| 18-19 | 1,258,753 | 1.5 | 8.0 | 10.2 | 54.0 | 1.6 | 5.2 | 1.6 | 80.8 | 19.2 | 23.2 | 14.5 | 16.8 |
| 20-24 | 3,487,863 | 0.8 | 11.1 | 8.8 | 48.1 | 1.0 | 5.1 | 3.6 | 76.0 | 24.0 | 21.2 | 13.6 | 18.4 |
| 25-29 | 3,801,409 | 0.9 | 12.7 | 6.9 | 48.6 | 0.7 | 4.9 | 5.4 | 77.6 | 22.4 | 19.2 | 10.9 | 17.5 |
| 30-34 | 3,807,954 | 1.3 | 17.0 | 5.7 | 50.9 | 0.6 | 5.3 | 7.1 | 86.3 | 13.7 | 18.7 | 7.4 | 12.3 |
| 35-39 | 3,733,642 | 1.7 | 20.7 | 4.0 | 50.2 | 0.4 | 4.6 | 8.6 | 89.0 | 11.0 | 18.0 | 5.8 | 10.3 |
| 40-44 | 3,414,297 | 3.1 | 53.4 | 1.3 | 23.6 | 0.1 | 2.4 | 11.9 | 95.4 | 4.6 | 19.4 | 3.3 | 5.3 |
| 45-49 | 3,715,812 | 2.6 | 53.6 | 1.4 | 17.5 | 0.2 | 2.7 | 14.0 | 90.9 | 9.1 | 16.6 | 4.0 | 8.1 |
| 50-54 | 3,907,461 | 2.0 | 54.0 | 0.8 | 14.7 | 0 | 0.3 | 25.4 | 95.4 | 4.6 | 15.1 | 1.7 | 3.9 |
| 55-59 | 3,670,651 | 1.8 | 49.6 | 0.6 | 15.6 | 0.0 | 0.1 | 32.9 | 99.2 | 0.8 | 13.3 | 0.5 | 1.4 |
| 60-64 | 3,111,835 | 1.4 | 39.0 | 0.4 | 15.1 | 0.0 | 0.1 | 45.7 | 99.9 | 0.1 | 11.1 | 0.0 | 0.1 |
| 65-69 | 2,796,740 | 0.7 | 22.1 | 0.2 | 11.0 | 0.0 | 0.0 | 65.4 | 98.5 | 1.5 | 7.8 | 0.2 | 1.3 |
| 70-74 | 2,779,326 | 0.1 | 12.6 | 0.6 | 7.2 | 0.0 | 0.0 | 79.8 | 99.6 | 0.4 | 6.3 | 0.0 | 0.3 |
| 75-79 | 1,940,686 | 0 | 3.3 | 0.4 | 3.0 | 0.0 | 0.0 | 93.9 | 99.2 | 0.8 | 6.6 | 0.0 | 0.0 |
| 80-84 | 1,439,913 | 0 | 4.1 | 1.0 | 11.0 | 0.0 | 0.0 | 84.4 | 99.5 | 0.5 | 7.3 | 0.1 | 0.4 |
| 85-89 | 879,778 | 0.9 | 4.9 | 0 | 11.5 | 0.0 | 0.0 | 82.7 | 98.2 | 1.8 | 10.2 | 0.2 | 0.6 |
| 90+ | 517,273 | 1.4 | 9.7 | 0 | 13.1 | 0.0 | 0.0 | 75.0 | 97.8 | 2.2 | 15.5 | 0.7 | 1.5 |

# Statistical analysis of survey results

We performed logistic regression analyses of risk mitigation behaviours from both surveys. We implemented four models, one for each of the following outcome variables: home testing kit use (Fig S1A), working from home (Fig S1B), using a face covering (Fig S1C) and limiting social contacts (Fig S1D). Due to high levels of vaccination in both cohorts we did not use vaccination as an outcome variable. In each model, we controlled for age (categories: < 30 years (reference), 30 – 39 years, 40 – 49 years, 50-59 years, 60 – 69 years, 70 – 79 years), gender (female (reference)/male) and survey cohort (ALSPAC (reference) /TwinsUK-Biobank). We excluded the small number of respondents who did not call within these demographic categories from the analysis.

The results in the main paper figure 1 are supported by the model estimates. For all outcomes variables apart from limiting social contacts, the TwinsUK-Biobank cohort reported higher levels of risk mitigation behaviours. Intention to use home testing kits and work from home decreased with age (Figs S1A&S1B). Intention to use a face covering was most pronounced in respondents aged 50 – 69 years. Males were less likely to report intending to use home testing kits but more likely to work from home, compared to females. There was no substantial effect of gender on use of face coverings or limiting social contacts.


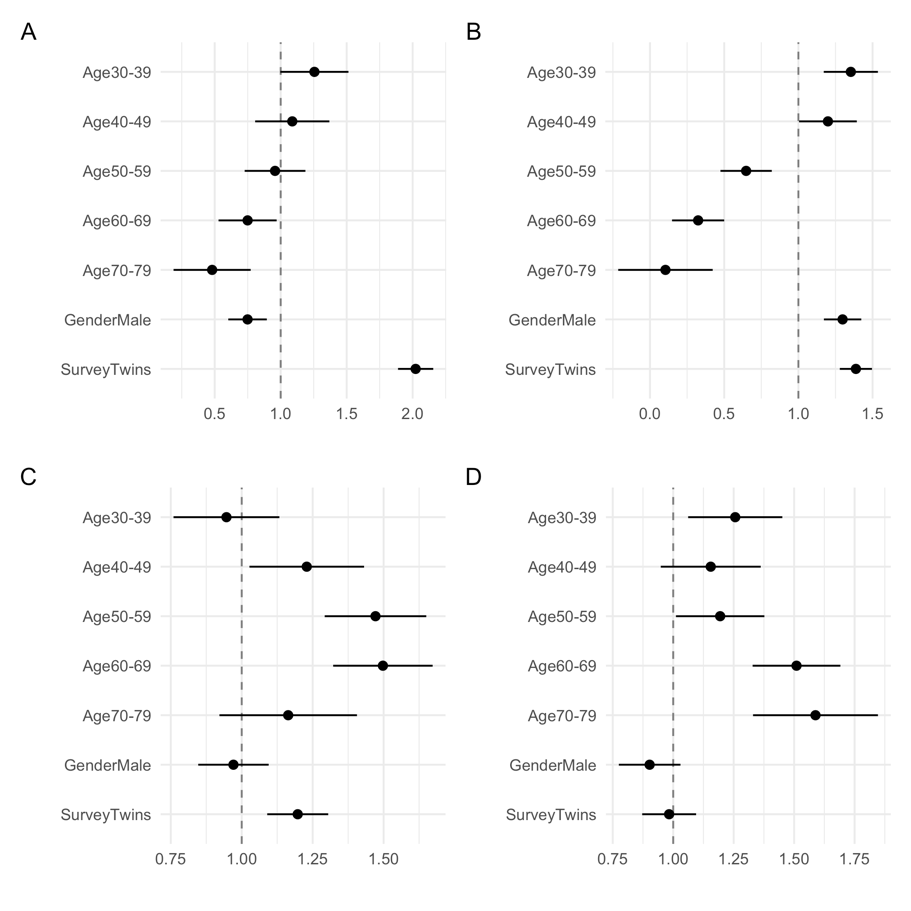


Figure S1: Results from the logistic regression models from the two prospective surveys. A) Use of testing kits; B) working from home; C) Use of face coverings; D) Limiting social contacts. The reference categories were under 30 years, female gender and the ALSPAC cohort.

# Sensitivity analysis of model output to key parameters

We explored the sensitivity of the model output to four key parameters: the vaccine escape exponent, the effectiveness of contact tracing, the effectiveness of COVID security and the sensitivity of home testing kits. We varied each parameter by +/- 20% from their baseline value used in the paper, with scenario 4 parameters. Figure S2 shows the resultant change in cumulative deaths. The parameter with the biggest impact was the vaccine escape exponent, leading a +/-0.25 absolute change in R and a +/- 1300 change in total deaths. A +/- 20% change in contact tracing effectiveness resulted in a +/-0.13 absolute change in R and a +/- 700 change in total deaths. A +/- 20% change in COVID security effectiveness resulted in a +/-0.08 absolute change in R and a +/- 300 change in total deaths. A +/- 20% change in home testing sensitivity resulted in a +/-0.06 absolute change in R and a +/- 200 change in total deaths (Figs S2 and S3).


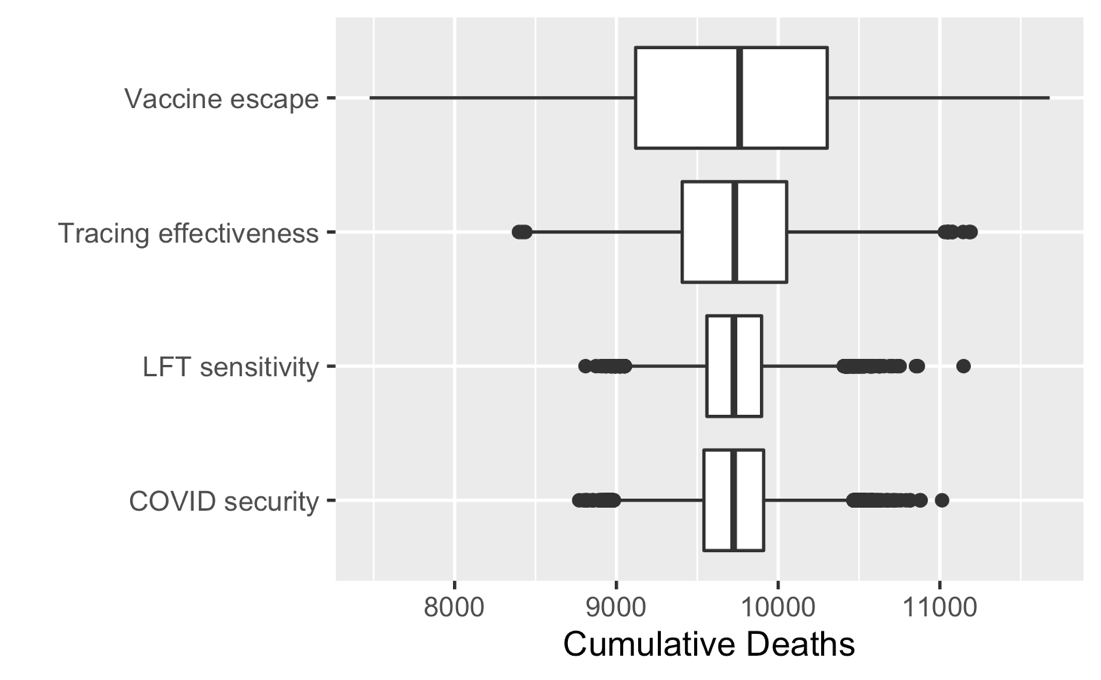


Figure S2: Sensitivity of the model output to four model parameters.


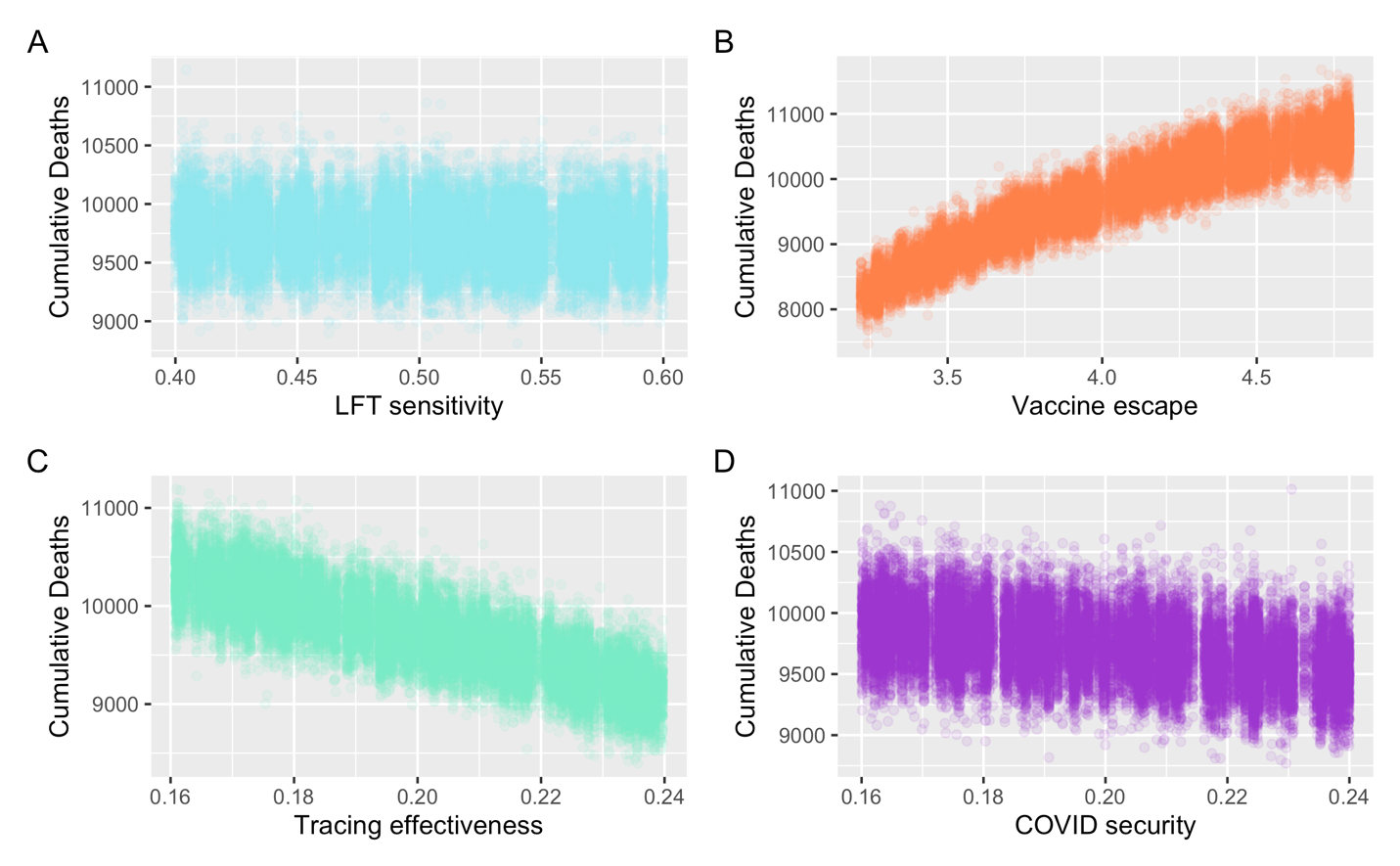


Figure S3: Exploration of the sensitivity of model output to four parameters.

# Survey questions from the ALSPAC survey

**ALSPAC Christmas survey questions** “Children of the 90s at Christmas”

**Making a difference to the pandemic – what are your plans for Christmas?**

For the questions, please think about the people you might be planning to spend time with indoors over the festive period. By this we mean **20^th^ December to 2^nd^ January inclusive**. This might include for example, visits to/from family members, get-togethers with friends at the pub or in a restaurant, an office party or other social event.

1. How many people in total in each of the following age groups do you think you will spend time with (for at least an hour) indoors over the festive period.

options are: none, 1-4, 5-9, 10+, don’t know

Pre-school children (aged under 5 years)

School/college aged children (aged 5 to 17 years)

Young Adults (aged 18-29 years)

Adults (aged 30-59 years)

Older adults (aged 60+ years)

2. How many other households, **excluding your own**, are you planning to spend time with (for at least an hour) indoors over the festive period?

None (I will stay with my household only)

1

2

3

4

5 or more

Don’t know

3. Will you be using any of the following precautionary measures during the festive period?

Yes/No/Don’t know for each question

Use home testing kits before meeting friends or relatives?

Work from home in the days before meeting friends or relatives?

Limit your contacts/exposure risk in the days before meeting friends or relatives?

Get vaccinated / receive the booster vaccine

Shop online, instead of visiting shops

Not using public transport

Increase indoor ventilation

Wear a mask in indoor spaces

4. Have you and the current members of your household been vaccinated against COVID-19?

Yes, all eligible household members have been vaccinated with at least one dose

Some eligible household members have been vaccinated with at least one dose

No household members have received a vaccination

I don’t know

5. How old are you?

< 30

30-39

40-49

50-59

60-69

70-79

80+

Prefer not to say

6. What is your gender?

Male

Female
 Non-Binary

Prefer not to say

7. Please tell us the **first part** of your postcode

e.g. if your postcode is BS1 9XX, please enter B in box 1, S in box 2 and 1 in box 3, leaving box 4 blank. If it is BS99 1XX, please enter B in box 1, S in box 2, 9 in box 3 and 9 in box 4.

Box 1

Box 2

Box 3

Box 4

8. Where will you be working **most of the time** in the lead up to the festive period? Please only tick one box

At Home

Healthcare setting such as hospital, doctor’s surgery, care home

Another setting where I will be in contact with other people (e.g. supermarket, office)

Another setting where my contact with other people will be limited (e.g. lorry driving,

Other

9. How will your patterns of activity change over the festive period compared to now?

I will meet more people

I will meet fewer people

I will meet approximately the same number of people.

I don’t know

10. Which generation of “Children of the 90s” study are you?

Parent

Original Child (born in 1990-1993) or partner of an original child

I’m not a participant in the study
